# Integrating a multimodal lifestyle intervention with medical food in prodromal Alzheimer’s disease: the MIND-AD_mini_ randomized controlled trial

**DOI:** 10.1186/s13195-024-01468-x

**Published:** 2024-05-30

**Authors:** Charlotta Thunborg, Rui Wang, Anna Rosenberg, Shireen Sindi, Pia Andersen, Sandrine Andrieu, Laus M. Broersen, Nicola Coley, Celine Couderc, Celine Z. Duval, Gerd Faxen-Irving, Göran Hagman, Merja Hallikainen, Krister Håkansson, Eija Kekkonen, Jenni Lehtisalo, Nicholas Levak, Francesca Mangialasche, Johannes Pantel, Anders Rydström, Anna Stigsdotter-Neely, Anders Wimo, Tiia Ngandu, Hilkka Soininen, Tobias Hartmann, Alina Solomon, Miia Kivipelto

**Affiliations:** 1 Division of Clinical Geriatrics, Department of Neurobiology, Care Sciences and Society, Center for Alzheimer Research QA32, Karolinska Institute, Karolinska Vägen 37 A, Solna, 171 64 Sweden; 2 Theme Inflammation and Aging, Karolinska University Hospital, Karolinska Vägen 37 A, Solna, Stockholm 171 64 Sweden; 3 Department of Physical Activity and Health, the Swedish School of Sport and Health Sciences, Stockholm, SE-114 86 Sweden; 4 Wisconsin Alzheimer’s Disease Research Center, School of Medicine and Public Health, University of Wisconsin, 600 Highland Ave J5/1 Mezzanine, Madison, WI 53792 USA; 5 Department of Neurology, Institute of Clinical Medicine, University of Eastern Finland, Yliopistonranta 8, Kuopio, 70210 Finland; 6 The Ageing Epidemiology (AGE) Research Unit, School of Public Health, Imperial College London, St Mary’s Hospital, Norfolk Place, London, W2 1PG UK; 7 Department of Clinical Epidemiology and Public Health, CHU de Toulouse, and Aging Research Team, CERPOP Inserm UMR 1295, Jules Guesde, Toulouse, 31000 France; 8 Danone Nutricia Research, Utrecht, Netherlands; 9 German Institute for Dementia Prevention (DIDP), Saarland University, Homburg, 66424 Germany; 10 Institute of General Practice, Goethe University Frankfurt a.M, Frankfurt, 60590 Germany; 11 Population Health Unit, Department of Public Health and Welfare, Finnish Institute for Health and Welfare, Mannerheimintie 166, P.O. Box 30, Helsinki, Finland; 12 Department of Social and Psychological Studies, Karlstad University, Karlstad, 651 88 Sweden; 13 Engineering Psychology, Luleå University of Technology, Luleå, 971 87 Sweden; 14 Department of Caring Sciences, Faculty of Health and Occupational Studies, Gävle, 801 76 Sweden; 15 Department of Experimental Neurology, Medical Faculty, Saarland University, Homburg, 66424 Germany; 16 Institute of Public Health and Clinical Nutrition, University of Eastern Finland, Yliopistonranta 8, Kuopio, 70210 Finland; 17 Stockholm Gerontology Research Center, Tomtebodavägen 18a, Solna, 171 65 Sweden; 18 IHU HealthAge, Toulouse, 31059 France

**Keywords:** Alzheimer’s disease, Lifestyle intervention, Multimodal intervention, Adherence, Medical food, Prevention, Randomized controlled trial

## Abstract

**Background:**

The Finnish Geriatric Intervention Study to Prevent Cognitive Impairment and Disability (FINGER) showed cognitive benefits from a multidomain lifestyle intervention in at-risk older people. The LipiDiDiet trial highlighted benefits of medical food in prodromal Alzheimer’s disease (AD). However, the feasibility and impact of multimodal interventions combining lifestyle with medical food in prodromal AD is unclear.

**Methods:**

MIND-AD_mini_ was a 6-month multinational (Sweden, Finland, Germany, France) proof-of-concept randomized controlled trial (RCT). Participants were 60–85 years old, had prodromal AD (International Working Group-1 criteria), and vascular/lifestyle risk factors. The parallel-group RCT had three arms: multimodal lifestyle intervention (nutritional guidance, exercise, cognitive training, vascular/metabolic risk management and social stimulation); multimodal lifestyle intervention + medical food (Fortasyn Connect); and regular health advice/care (control). Participants were randomized 1:1:1 (computer-generated allocation at each site). Outcome evaluators were blinded to randomization. Primary outcome was feasibility of the multimodal intervention, evaluated by recruitment rate during a 6-month recruitment phase, overall adherence in each intervention arm, and 6-month retention rate. Successful adherence was pre-specified as attending ≥ 40% of sessions/domain in ≥ 2/4 domains (lifestyle intervention), and consuming ≥ 60% of the medical food (lifestyle intervention + medical food). The secondary outcomes included adherence/participation to each intervention component and overall adherence to healthy lifestyle changes, measured using a composite score for healthy lifestyle. Cognitive assessments were included as exploratory outcomes, e.g. Clinical Dementia Rating scale.

**Results:**

During September 2017-May 2019, 93 individuals were randomized (32 lifestyle intervention, 31 lifestyle + medical food, and 30 control group). Overall recruitment rate was 76.2% (64.8% during the first 6 months). Overall 6-month retention rate was 91.4% (lifestyle intervention 87.5%; lifestyle + medical food 90.3%; control 96.7%). Domain-specific adherence in the lifestyle intervention group was 71.9% to cognitive training, 78.1% exercise, 68.8% nutritional guidance, and 81.3% vascular risk management; and in the lifestyle + medical food group, 90.3% to cognitive training, 87.1% exercise, 80.7% nutritional guidance, 87.1% vascular risk management, and 87.1% medical food. Compared with control, both intervention arms showed healthy diet improvements (β_Lifestyle×Time_ = 1.11, *P* = 0.038; β_Lifestyle+medical food×Time_ = 1.43, *P* = 0.007); the lifestyle + medical food group also showed vascular risk reduction (*P* = 0.043) and less cognitive-functional decline (*P* < 0.05, exploratory analysis). There were 5 serious adverse events (control group: 1; lifestyle intervention: 3; lifestyle + medical food: 1) unrelated to interventions.

**Conclusions:**

The multidomain lifestyle intervention, alone or combined with medical food, had good feasibility and adherence in prodromal AD. Longer-term cognitive and other health benefits should be further investigated in a larger-scale trial.

**Trial registration:**

ClinicalTrials.gov NCT03249688.

**Supplementary Information:**

The online version contains supplementary material available at 10.1186/s13195-024-01468-x.

## Background

Preventive interventions for dementia and Alzheimer's disease (AD) are a key priority [1]. AD includes preclinical, prodromal, and dementia stages [2, 3]. Preclinical AD is largely asymptomatic, while prodromal AD comprises mild cognitive impairment and amyloidopathy-related biomarkers [3]. To ensure optimal effects, clinical trials are increasingly focusing on prodromal AD [4–6], including multimodal non-pharmacological intervention trials targeting multiple risk factors and disease mechanisms simultaneously [7, 8]. Nutritional approaches have been highlighted in this stage, e.g. the medical food Fortasyn Connect in the LipiDiDiet trial [9]. LipiDiDiet findings indicated that medical food can benefit patients with prodromal AD by slowing down cognitive and functional decline, brain atrophy, and disease progression [9, 10]. Earlier-stage interventions in at-risk older adults have also reported cognitive and other health-related benefits, e.g. a 2-year multidomain lifestyle intervention (diet, exercise, cognitive training, social activities, and vascular risk monitoring) tested in the Finnish Geriatric Intervention Study to Prevent Cognitive Impairment and Disability (FINGER) [11–14]. As prodromal AD is a critical stage for intervention, a multimodal approach combining multidomain lifestyle intervention and medical food may have joint effects and further improve benefits. Nevertheless, the feasibility and impact of complex lifestyle interventions, alone or in combination with medical food, is unclear among patients with prodromal AD.

The MIND-AD_mini_ trial was designed with the primary objective of testing the feasibility of a multimodal intervention (adapted FINGER-based lifestyle intervention, with or without medical food) in prodromal AD [15]. Secondary outcomes included adherence to specific intervention domains (in the 2 intervention groups) and adherence to healthy lifestyle changes. Additional analyses were performed to investigate intervention effects on cognitive-functional measures.

## Methods

### Study design and participants

The MIND-AD_mini_ trial was a 6-month proof-of-concept multicentre, randomized controlled parallel-group trial (three arms). A detailed trial protocol has been published previously [15]. Participants were recruited from memory clinics in Stockholm, Sweden, and Toulouse, France, via advertisement in Frankfurt Germany, and from the university hospital neurology clinic and previous research cohorts in Kuopio, Finland. Pre-screening was performed for some of the inclusion criteria where information was available. Inclusion criteria were: age 60–85 years; Mini-Mental State Examination (MMSE) ≥ 24 points; availability of a study partner; prodromal AD according to International Working Group-1 (IWG-1) criteria including episodic memory impairment and underlying AD pathology [2, 16]; and an index indicating potential for lifestyle improvement. We defined episodic memory impairment as -1 SD on at least 2 out of 8 tests, at least 1 being a memory test: Free and Cued Selective Reminding Test (FCSRT) delayed free recall ≤ 8, FCSRT free recall-learning ≤ 22, Wechsler Memory Scale-revised (WMS-R) story delayed recall ≤ 75%, WMS-R delayed recall figures ≤ 75%, Trail Making Test (TMT) A ≥ 60 s, TMT-B ≥ 60 s, symbol digit substitution test ≥ 35 (120 s), category fluency ≤ 16 (60 s). AD pathology was defined as having ≥ 1 abnormal cerebrospinal fluid (CSF, CSF β-amyloid (1–42/1–40) × 10 ratio < 1 and/or total-tau and/or phospho-tau and/or β-amyloid 42 based on local cut-offs) or neuroimaging biomarker (Scheltens medial temporal lobe atrophy score of at least 1and/or abnormal FDG-PET and/or PiB-PET compatible with AD type changes) [15]. A score of 2 or above was additionally required on a lifestyle index based on physical inactivity, unhealthy dietary habits, hypertension, diabetes, sleep disturbances, depressive symptoms or psychological stress symptoms [15].

Exclusion criteria were: dementia diagnosis; conditions affecting safe intervention engagement (e.g., exercise); concomitant severe diseases (e.g., recent history of myocardial infarction or cancer); major depressive disorder; MRI/CT scan indicating stroke, intracranial bleeding, mass lesion or normal pressure hydrocephalus; intake of vitamin B6, B12, folic acid, vitamin C and/or E supplements > 200% recommended daily intake unless prescribed by a physician; use of omega-3 preparations (> 500 mg EPA + DHA per day); alcohol or drug abuse; severe loss of vision or communicative ability; conditions preventing cooperation as judged by the study physician; and concomitant participation in any intervention trial.

### Randomization and masking

Participants were randomized to lifestyle intervention, lifestyle intervention + medical food, or control group receiving regular health advice in 1:1:1 ratio in blocks of six (computer generated allocation, two individuals randomly allocated to each group) at each of the four sites after screening by the study nurse. Outcome evaluators were blinded to the randomization group and were not involved in intervention activities. Similar to the FINGER trial, group allocation was not actively disclosed to participants, and participants were instructed not to discuss the intervention with outcome evaluators.

### Intervention

The control group received regular health advice. All participants met the study nurse at screening, baseline, and 6 months after randomisation for health measurements (e.g., blood pressure, weight and BMI, and hip and waist circumference). All participants met the study physician at screening and 6-month visits for detailed medical history and physical examination. At baseline, the study nurse gave all participants (control and intervention groups) oral and written information and advice on healthy diet and physical, cognitive, and social activities that are beneficial for management of vascular risk factors and disability prevention. In the case of clinically relevant abnormal blood tests (samples collected at baseline and 6-months), participants were provided with information and advice to contact primary health care or a referral to primary health care. All participants had a chance to contact the study nurse by telephone or e-mail when needed.

The multimodal lifestyle intervention group received a FINGER-based intervention program adapted for individuals with prodromal AD [15]. Intervention duration was 6 months. Stepwise introduction of intervention domains was used to promote adherence. Diet and physical activity started during the first month, cognitive training started during the second month following randomization. Given that intervention domains had both individual and group sessions, there could be a delay of up to 1–2 months before all domains were introduced. Intervention sessions and schedule were aligned among study centers to ensure similar intervention content and intensity for all participants, while leaving room for some local adaptations and flexibility [15]. Interventions were delivered in university facilities (Germany), and university hospital and private commercial gym facilities (Sweden, Finland, and France).

The FINGER-based lifestyle intervention combined five domains. *Nutritional guidance*, following the Nordic Nutrition Recommendations (NNR) 2012 or adapted to the country’s nutritional recommendations (France and Germany) [17], was provided by a registered dietitian/nutritionist through 3 individual counselling sessions and 3–4 group sessions. *The physical exercise training program*, supervised by a physiotherapist or personal trainer, was tailored to each participant’s fitness level and included cardiovascular endurance and progressive strength training [15, 18–20], Training sessions were conducted twice/week (60 min/session) with groups of 4–5 participants. *Cognitive training* included group and individual sessions. The 2–3 group sessions (60–75 min/session), led by psychologists or occupational therapists, included general information about neurocognitive disorders, coping and reasoning strategies, introducing the cognitive training program and instructing its use. Individual training sessions consisted of computer-based training at home or study site (twice/week, 15–30 min/session). The cognitive training program was a web-based, in-house developed computer program including several tasks adapted from protocols previously used in the FINGER trial [11, 15]. *Social activities* were stimulated through group sessions within the intervention domains (exercise, nutrition, and cognitive training), designed to facilitate open discussions and interactions among participants. *Monitoring and management of vascular/metabolic risk factors*, following national evidence-based guidelines, comprised one additional study nurse visit at 3 months, for blood pressure, weight and BMI, hip and waist circumference measurements, and further recommendations for lifestyle management, including smoking cessation. If medication initiation or adjustments were needed, the study physician either wrote a prescription or referred the participant to regular healthcare, as per local procedures.

The multimodal lifestyle + medical food intervention group received all lifestyle intervention domains mentioned above, plus the study product Fortasyn Connect (Souvenaid™), a 125 ml once-a-day milk-based drink including a complex nutrient combination (long-chain omega-3 fatty acids docosahexaenoic acid (DHA) and eicosapentaenoic acid (EPA), uridine monophosphate, choline, vitamins B12, B6, C, E, and folic acid, phospholipids, and selenium) designed to enhance efficacy over what can be achieved by individual nutrients. Danone Nutricia Research provided this study product for the MIND-AD_mini_ trial.

### Outcome measures

*The primary outcome* was feasibility of the multimodal intervention measured by recruitment rate, retention rate, and overall intervention adherence (in the 2 intervention arms). *Recruitment rate* was calculated by dividing the number of randomised participants by the number of potentially eligible participants who were invited to participate during a 6-month recruitment phase. Due to study sites having different start dates, recruitment rate was calculated considering the initial 6-month period at each site. A recruitment rate of ≥ 50% was pre-specified as successful [15]. *Retention rate* was calculated as the proportions of participants who completed the 6-month trial period. A successful retention rate was pre-specified as ≤ 35% of participants dropping out. *Overall intervention adherence* was calculated in the 2 intervention arms (multimodal lifestyle ± medical food) as a composite measure of participation in different intervention domains. The number of attended sessions was divided by the total number of sessions offered to the participants. Successful adherence to the lifestyle intervention was pre-specified as attending ≥ 40% of sessions/domain in at least 2/4 domains (exercise, nutrition, cognitive training and monitoring and management of vascular/metabolic risk factors); for the lifestyle + medical food arm, consuming ≥ 60% of the medical food study product was additionally required.

*The secondary outcomes* included adherence to specific intervention domains (in the 2 intervention groups), and adherence to healthy lifestyle changes (all participants). *Adherence to specific intervention domains* was computed as follows: *Nutrition adherence* was defined as attending at least 2 out of 3 of the group sessions, and 2 out of 3 of the face-to-face dietary counselling sessions. *Exercise adherence* was defined as attendance in at least 40% of the twice per week offered group-based gym sessions. *Cognitive training adherence* was determined by attendance in at least 2 out of the 3 group sessions, and automatic recordings of computer program use, i.e., number of completed training blocks divided by 48 (maximum offered number). *Adherence to monitoring and management of vascular/metabolic risk factors* was defined as attending 3- and 6-month meetings with the study nurse for cardiovascular health measurements. *Medical food adherence* was defined as consuming ≥ 60% of the medical food product based on diary information, i.e., bottles consumed divided by bottles delivered for each participant.

*Adherence to Specific healthy lifestyle changes* was calculated in all trial participants based on four domains: healthy dietary intake, physical activity, cognitive and social activities (considered as a single domain), and cardiovascular risk burden, measured at baseline and month 6. *Healthy dietary intake* was based on a modified Mediterranean Diet Adherence Screener (MEDAS) score calculated from the food frequency questionnaire, with a higher score indicating a healthier dietary intake [21]. *Physical activity* was measured actigraph-measured percentage of daily time spent on moderate to vigorous physical activity (ActiGraph GT3X, Pensacola, FL, USA); for 28 participants with missing actigraph data, the Swedish National Board of Health and Welfare self-reported physical activity questionnaire was used instead. *Cognitive and social activities* were quantified as self-reported engagement in different types of cognitive (i.e. studying, writing, crossword puzzles, hand crafts, course participation) and social activities (i.e. volunteering, engagement in a club/association, taking care of children, playing card and board games) [12]. Engagement in each activity was rated using a 7-point Likert scale of frequencies ranging from a daily to never, with higher score indicating more engagement. *Overall cardiovascular risk burden* was measured using the FINRISK score including age, sex, serum total cholesterol, systolic blood pressure, HDL-C, smoking status, and diabetes, with a higher score indicating higher cardiovascular risk [22]. Each participant’s overall cardiovascular risk score was divided by the overall cardiovascular risk score calculated for a sex and age-matched person without any cardiovascular risk factors, as previously defined [22]. *Overall adherence to healthy lifestyle changes* was calculated as a composite healthy lifestyle score, based on the specific healthy lifestyle changes, in all trial participants at baseline and month 6, by adapting a method previously used in the FINGER trial [23]. A score from 0 to 2 (with higher score indicating healthier lifestyle) was assigned to each tertile of the healthy dietary intake score, physical activity level, cognitive and social activities (lowest tertile = 0, middle = 1, highest = 2), and cardiovascular risk burden (lowest tertile = 2, middle = 1, highest = 0). The composite healthy lifestyle score was calculated as the sum of tertile scores in all four domains (ranges from 0 to 8).

### Exploratory assessments

The Clinical Dementia Rating Scale (CDR) was used to evaluate cognitive-functional level at baseline and 6 months [24, 25]. The CDR-Sum of Boxes (SOB) score was calculated by summing the scores from each domain. An increased score on the CDR-SOB or global CDR score indicates more severe cognitive impairment. The range of the CRD score is 0–3 points, and the range of the CDR-SOB score is 0–18 points.

### Statistical analysis

Because primary outcome measures focused on feasibility, formal sample size calculations were not performed. Analyses included all randomized participants, except for adherence to intervention measures which were limited to all participants randomized to the 2 intervention arms. Baseline characteristics were compared between trial arms using chi-square test (categorical variables) or one-way analysis of variance/Kruskal–Wallis test (continuous variables). Recruitment, retention and intervention adherence rates are reported using descriptive statistics. Differences from baseline to month 6 between trial arms regarding adherence to healthy lifestyle changes (overall and per domain) were investigated using linear mixed-effects models. This analysis adjusted for the cluster effect by capturing the correlation among participants within each site and accounting for individual variations within clusters and groups. We report estimates and 95% confidence intervals (CIs) for two-way interactions between randomization arms (control, lifestyle, and lifestyle + medical food) and time (baseline vs. month 6) in the linear mixed-effect models.

To evaluate differences between intervention arms (lifestyle or lifestyle + medical food) and the control arm in changes in the CDR-SOB and global CDR scores, we applied the generalized estimating equations with an ordinal logit model, with a robust variance estimator (cluster by each participant). We considered the CDR-SOB and global CDR scores as “ordinal” outcome measures because their mutually exclusive categories can be ordered by severity of cognitive-functional symptoms [26]. We estimated odds ratio (ORs) and 95% CI of two-way interactions between randomization arms (control, lifestyle, and lifestyle + medical food) and time (baseline v.s. 6 month) in the models.

All statistical analyses were performed in STATA 17.0 software by an independent statistician who was blinded to randomization.

## Results

**Fig. 1:**
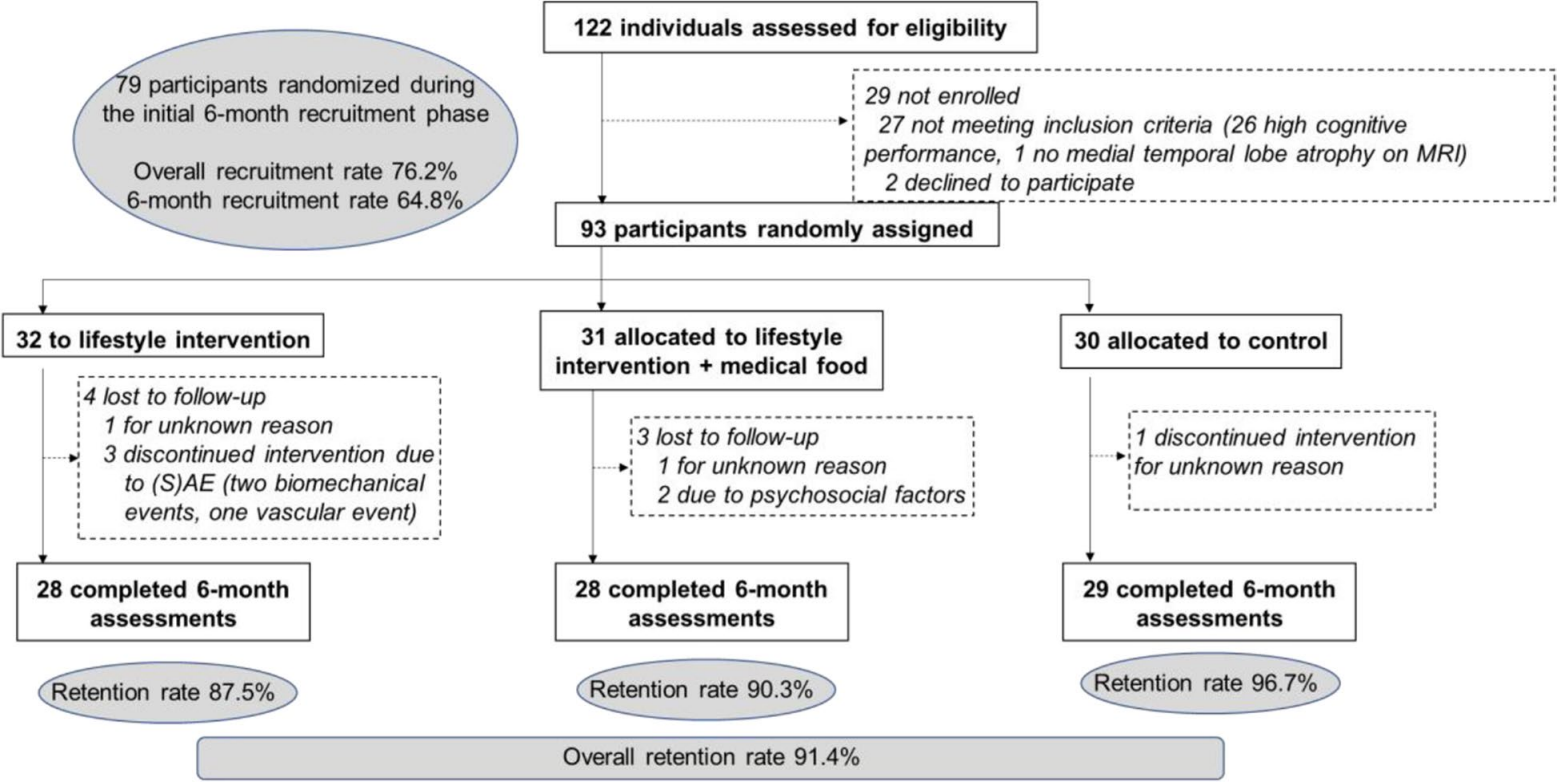
CONSORT flowchart

The trial CONSORT flowchart is shown in Fig. 1. Recruitment occurred during different time periods at the four sites: October 2017 to April 2018 in Sweden, November 2017 to May 2018 in Finland, March 2018 to July 2018 in Germany, and March 2018 to May 2019 in France. One-hundred thirty-four potential participants were pre-screened and 122 were assessed for eligibility and 93 of them were assigned to different group (lifestyle intervention, lifestyle intervention + medical food, and control). Overall recruitment rate was 76.2% (64.8% during the initial 6-month recruitment phase). Eight (8.6%) participants dropped out (Fig. 1). Eighty-five participants (lifestyle intervention *n* = *28*; lifestyle intervention + medical food; *n* = *28*, or control; *n* = *29*) completed the interventions. Overall retention rate was 91.4% (87.5% in the lifestyle intervention arm, 90.3% in the lifestyle + medical food intervention arm, and 96.7% in the control arm).

The 3 randomization arms were similar at baseline (Table 1). Population mean age (SD) was 72.9 (6.2) years, 50 participants (53.7%) were women, mean education (SD) was 12.8 (3.6) years, and mean MMSE (SD) 27.7 (1.6) points.

**Table 1:** Baseline characteristics of all randomized participants

	**Multimodal lifestyle intervention (*n* = 32)**	**Multimodal lifestyle intervention + Medical food (*n* = 31)**	**Control (regular care) (*n* = 30)**	***P*-value**
**Sites, n (%)**				
Germany	8 (25.00)	8 (25.81)	8 (26.67)	
Finland	10 (31.25)	10 (32.26)	10 (33.33)	
Sweden	12 (37.50)	12 (38.71)	12 (44.00)	
France	2 (6.25)	1 (3.23)	0	0.936
**Age (years)**				
Median (min–max)	72.50 (60–83)	73.00 (61–84)	74.00 (61–85)	0.718
**Sex, no (%)**				
Men	11 (34.38)	16 (51.61)	16 (53.33)	
Women	21 (65.63)	15 (48.39)	14 (46.67)	0.249
**Education level, n (%)**				
Primary school	4 (12.50)	4 (12.90)	2 (6.67)	
Secondary school	12 (37.50)	6 (19.35)	10 (33.33)	
University	16 (50.00)	21 (67.74)	18 (60.00)	0.492
**Healthy dietary intake (MEDAS score)**				
Median (IQR)	6 (4–7)	6 (5–8)	6 (5–7)	0.774
**Physical activity (% daily moderate to vigorous physical activity)**				
**Median (IQR)**	1.91 (1.11–3.27)	2.57 (1.88–2.85)	1.81 (1.18–3.19)	0.500
**Engagement in cognitive and social activities**				
Median (IQR)	20.05 (14.55–26.65)	20.05 (17.20–26.10)	21.35 (15.33–26.10)	0.809
**Overall cardiovascular risk burden (based on FINRISK score)**				
Median (IQR)	1.01 (0.76–1.40)	1.30 (0.94–1.89)	1.26 (0.95–1.94)	0.258
**Composite healthy lifestyle score**				
Median (IQR)	5 (3–5)	4 (3–5)	3 (2–5)	0.630
**Global CDR score**				
Median (Range)	0.5 (0–0.5)	0.5 (0–0.5)	0.5 (0–0.5)	0.924
**CDR-SOB**				
Median (IQR)	1 (0.5–1.75)	1 (0.5–2.5)	0.5 (0.5–1.5)	0.676
**Mini-Mental State Examination**				
Median (IQR)	28 (26–29)	28 (27–29)	28 (27–29)	0.755

*IQR* Interquartile range, *MEDAS* Mediterranean Diet Adherence Screener, *CDR-SOB* Clinical Dementia Rating Scale Sum of Boxes. Data were missing for *n* = 4 participants for engagement in cognitive and social activities; *n* = 5 for physical activity; *n* = 4 for MEDAS score; *n* = 22 for the FINRISK score; and *n* = 26 for the composite healthy lifestyle score (missing FINRISK and composite healthy lifestyle scores were due to missing systolic blood pressure recordings at baseline because of a technical issue at one of the sites in the beginning of the study)

All *p*-values > 0.2

Overall adherence to the intervention and adherence to each intervention domain are shown in Table 2. In the lifestyle intervention arm, 78.1% adhered to at least 2 out of 4 intervention domains, with domain-specific adherence 68.8% for nutrition guidance, 78.1% for physical exercise, 71.9% for cognitive training, and 81.3% for vascular care domain. In the lifestyle + medical food arm, 87.1% of participants were overall adherent, with domain-specific adherence 80.7% for nutrition guidance, 87.1% for physical exercise, 90.3% for cognitive training, 87.1% for vascular care, and 87.1% for medical food.

**Table 2:** Overall intervention adherence, and adherence to specific intervention domains

	**Multimodal lifestyle intervention (*n* = 32)**	**Multimodal lifestyle intervention + Medical food (*n* = 31)**
**Overall intervention adherence, n (%)**	**25 (78.1)**	**27 (87.1)**
**Adherence to each intervention domain, n (%)**		
**Nutrition**		
Attendance in ≥ 2 group sessions and ≥ 2 individual face to face meetings	22 (68.8)	25 (80.7)
**Exercise**		
Attendance in ≥ 40% of the offered group-based gym sessions^a^	25 (78.1)	27 (87.1)
**Cognitive training**		
Attendance in ≥ 2 group sessions and number of completed training blocks divided by 48 (maximum offered number)^b^	23 (71.9)	28 (90.3)
**Vascular care**		
Attendance in the 3- and 6-month meetings with the study nurse	26 (81.3)	27 (87.1)
**Medical food**		
Consuming ≥ 60% Souvenaid (bottles consumed divided by bottles delivered for each participant)	--	27 (87.1)

The different intervention domains were introduced gradually to avoid over-burdening participants. To stimulate the social component, the group sessions did not start until enough participants (4–5) were randomized to form a small group, i.e. there could be a delay of up to 1–2 months from baseline until the start of group sessions

^a^Calculated based on the number of sessions offered to each participant. During the 6-month trial, the amount of offered sessions could differ slightly due to public holidays and vacations

^b^In total 5 participants had missing data (automatic recordings)

**Fig. 2:**
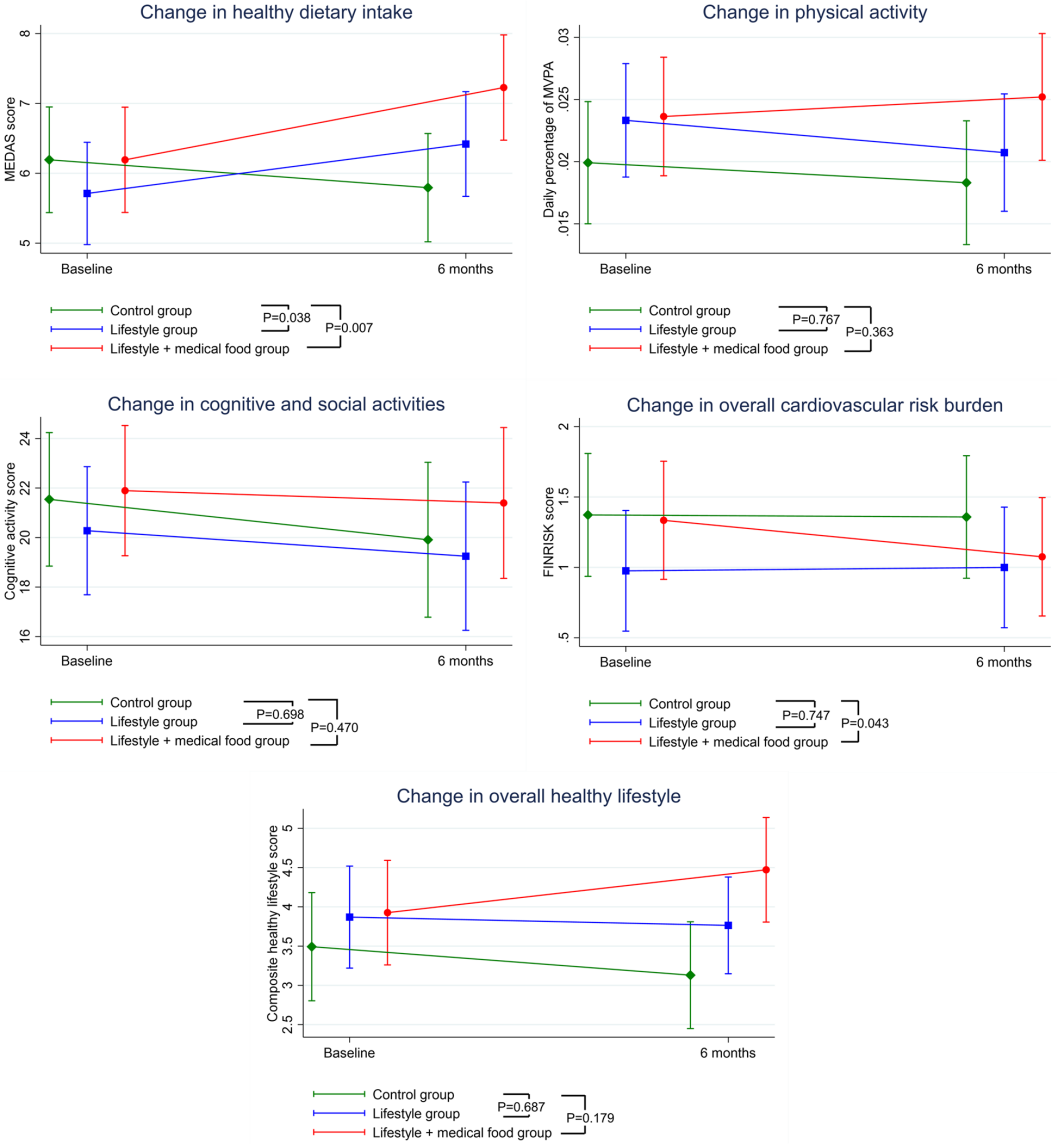
Adherence to healthy lifestyle changes from baseline to month 6 in all randomized participants. When compared to the lifestyle intervention group, the lifestyle intervention + medical food group showed significant improvement in cardiovascular risk burden (*P* = 0.016), but no differences were observed in changes for healthy dietary intake (*P* = 0.533), physical activity (*P* = 0.215), cognitive and social activities (*P* = 0.726), or overall healthy lifestyles (*P* = 0.338)

Adherence to healthy lifestyle changes from baseline to month 6 in all randomized participants is shown in Fig. 2. Healthy dietary intake increased significantly in both the lifestyle intervention and the lifestyle + medical food arms compared with control. The estimated mean difference (95% CI) in MEDAS score change was 1.11 (0.06–2.15) for the lifestyle intervention versus control arm, and 1.43 (0.39–2.48) for the lifestyle + medical food versus control arm. The overall cardiovascular risk burden decreased significantly in the lifestyle + medical food intervention arm compared with control, with an estimated mean difference (95% CI) of -0.24 (-0.48 to -0.01) in FINRISK score change. The FINRISK score changes was not significantly different between the lifestyle intervention and control arms. There were no statistically significant differences between either intervention arm and control concerning change in time spent daily on moderate-to-vigorous physical activity, engagement in cognitive and social activities, or the composite healthy lifestyle score (Fig. 2).

The likelihood of cognitive-functional decline (change in global CDR score and CDR-SOB) from baseline to month 6 are shown in Table 3. Overall, cognitive-functional level decreased during 6 months across all arms, with OR (95% CI) 3.19 (1.94–5.26) for an increase in CDR-SOB score. The lifestyle + medical food intervention arm had a significantly lower likelihood for decreasing cognitive-functional level (i.e. increasing CDR-SOB) compared with control, with OR (95%) 0.44 (0.21–0.92). The change in CDR-SOB was not significantly different between the lifestyle intervention and control arms. There were no statistically significant differences between either intervention arm or control concerning change in global CDR score.

**Table 3:** Likelihood of cognitive-functional decline during the 6-month trial period

	**CDR-SOB score**	**Global CDR score**
	OR (95% CI)	
**Likelihood of decline across all trial arms**	3.19 (1.94–5.26)^**^	2.04 (0.66–6.30)
**Likelihood of decline compared with the control arm**		
Lifestyle intervention arm	1.41 (0.70–2.85)	2.59 (0.39–16.98)
Lifestyle + medical food arm	0.44 (0.21–0.92)^*^	0.54 (0.13–2.23)

*CDR* Clinical Dementia Rating Scale, *CDR-SOB* Clinical Dementia Rating Scale Sum of Boxes

^*^0.01 < *P* < 0.05; ^**^P < 0.01

A summary of adverse events in all randomized participants is shown in Table 4. The incidence of adverse events was highest in the control arm (81.3%), and lowest in the lifestyle intervention arm (45.2%). The most common adverse events (reported by at least 5 participants) were musculoskeletal, cardiovascular and respiratory symptoms. Only 5 participants experienced serious adverse events: 3 in the lifestyle intervention arm, leading to discontinuation (one with spinal stenosis, one with stroke, and one with nasal polyps surgery); 1 in the lifestyle + medical food arm (stroke); and 1 in the control arm (urinary tract infection). None of the serious adverse events were regarded as related to any of the intervention components. No participants died during the 6-month trial period.

**Table 4:** Summary of adverse events in all randomized participants

**All events (n people/N events)**	**Control (*n* = 30)**	**Multimodal lifestyle intervention (*n* = 32)**	**Multimodal lifestyle intervention + medical food (*n* = 31)**
***Adverse events***	15/25	9/11	12/22
Musculoskeletal and connective tissue disorders	6/6	3/3	5/5
Fall	1/1	0	1/1
Cardiac disorders	2/2	1/1	0
Vascular disorders	1/1	0	1/1
Respiratory, thoracic, and mediastinal disorders	1/1	3/3	2/2
Gastrointestinal disorders	4/4	2/2	3/3
Psychiatric disorders	2/2	0	2/4
Skin and subcutaneous tissue disorders	2/3	1/2	2/2
Renal and urinary disorders	1/1	0	3/3
Reproductive system and breast disorders	0	0	1/1
Dental and gingival conditions	1/1	0	0
General disorders and administration site conditions/Investigations	3/3	0	0
***Serious adverse events***	1/1	3/3	1/1
Musculoskeletal and connective tissue disorders	0	1/1	0
Vascular disorders	0	1/1	1/1
Respiratory, thoracic, and mediastinal disorders	0	1/1	0
Renal and urinary disorders	1/1	0	0

Data show number of participants / number of events. Some participants reported more than one event. (Serious) adverse events are presented by Medical Dictionary for Regulatory Activities preferred term. Adverse events were recorded at the 6-month visit in all participants, and also the 3-month visit in the two intervention arms. Participants were also asked if they had experienced any harm related to the study, such as stress or musculoskeletal pain

## Discussion

This study tested the feasibility of a 6-month multimodal intervention (adapted FINGER-based lifestyle intervention, with or without medical food) in older adults with prodromal AD. Criteria for success were exceeded for primary outcome measures: recruitment rate 76.2% versus pre-specified 50%; overall retention rate 91.4% versus pre-specified 65%; and overall intervention adherence rate 78.1%. To our knowledge, this is the first study showing that the combination of a multidomain lifestyle intervention and medical food is feasible in prodromal AD. Compared with control, the lifestyle + medical food arm also had significantly more improvement in healthy dietary intake, reduction in overall cardiovascular risk burden, and lower likelihood of cognitive-functional decline. The lifestyle intervention arm had significantly more improvement in healthy dietary intake, but was not different from the control arm regarding change in other lifestyle or cognitive-functional measures.

Finding effective and feasible interventions for individuals with prodromal AD is crucial as effective disease-modifying drugs are not yet widely available [2, 27]. In addition, many memory clinic patients with mild cognitive impairment will likely not be eligible for new anti-amyloid drugs [28]. Complex multimodal lifestyle interventions have shown benefit in older at-risk individuals without substantial impairment [7, 11]. This study shows that complex multimodal interventions are feasible, and may have lifestyle-related and possibly cognitive-functional benefits also in older adults who already have cognitive impairment. Adding a medical food component to the multidomain lifestyle intervention can be particularly important in prodromal AD [15]. While nutritional status is important for healthy brain aging and dementia risk reduction [29], dietary guidance may not be sufficient for patients with prodromal AD, who are frequently deficient in essential nutrients [30]. The lifestyle + medical food combination is also highly relevant as a step towards future intervention trials combining pharmacological and non-pharmacological interventions in prodromal AD [15].

The 6-month dropout rates were 13% in the lifestyle arm and < 4% in the lifestyle + medical food arm in patients with prodromal AD. In comparison, drop-out rates were 21% in the LipiDiDiet trial (2-year medical food intervention, prodromal AD) [10], 12% in the FINGER trial (2-year multidomain lifestyle intervention, at-risk older adults without substantial impairment) [13], and 22.5% in the MAPT trial (3-year multidomain lifestyle intervention, frail older general population) [31].

Previously reported domain-specific adherence rates for multidomain lifestyle interventions ranged from 47.2% to 92.9% in the FINGER trial, and from 53.5% to 71.5% in the MAPT trial [31]. In the MIND-AD_mini_ trial, overall intervention adherence was 78.1% for the lifestyle arm, and 87.1% for the lifestyle + medical food arm, with domain-specific intervention adherence rates ranging from 68.8% to 90.3%. The adherence rate to the medical food intervention component was 87%, similar to the 93.4% adherence reported in the LipiDiDiet trial [10]. Although the analytical sample was small in this pilot study, our findings suggest potentially increased adherence in interventions combining lifestyle with other medical treatment-like components. Possible reasons underlying this finding need to be investigated in future studies.

Outside Europe, reported domain-specific adherence rates for FINGER-based multidomain lifestyle interventions ranged from 33 to 100% in the 6-month SINGER trial (Singapore) [32] and from 94 to 100% in the 8-week SUPERBRAIN trial (South Korea) [33]. The adherence rate to the nutritional supplement in the Korean study (multinutrient drink) was 99.1% in the group also receiving lifestyle intervention and 83.7% in the group only receiving the supplement. Another pilot feasibility 6-month RCT in China with lifestyle intervention comprising cognitive training, mind–body exercise, and nurse-led risk factor modification reported a much lower recruitment rate (76% vs. 19%), a similar dropout rate (10% vs. 11%), and a comparable overall adherence rate in the multidomain lifestyle arm (78.1% vs. 89%) [34]. Differences in findings may be attributable to different inclusion criteria, study design, and definitions of successful feasibility and adherence in the Chinese study [35]. We observed a significant improvement in the dietary pattern compared to other lifestyle changes. The diet component was introduced as the first intervention domain, potentially allowing more time to detect efficient changes in dietary patterns compared to other intervention domains. Future studies are encouraged to validate significant lifestyle changes by engaging more participants and implementing longer follow-up periods. Also, the cognitive and social activities questionnaire did not cover activities that were part of the intervention. Since both intervention arms had frequent activities offered by the study, they may have had less time for other kinds of activities.

This study has several strengths, including multi-country setting, and comprehensive measurements of multiple lifestyle domains. Additionally, the use of device-based physical activity measurements by actigraphy captured objectively measured exercise data across multiple intensity levels at baseline and 6 months. The use of on-site cognitive training adapted for people with prodromal AD facilitated adherence to the training program. However, there are some limitations. The relatively small sample size and 6-month duration of this trial limit statistical power and the possibility to assess longer-term adherence, retention and effects on cognition and dementia incidence. The multi-national trial presented some challenges in achieving balanced recruitment rates across all sites, with some sites experiencing lower recruitment due to competitive inclusions between multiple ongoing clinical trials. This also resulted in a recruitment phase longer than the initially planned 6 months. The trial included a demographically limited population, largely comprising white participants from Sweden, Finland, Germany, and France. Recruitment, retention, and adherence may be different in other European or non-European populations, especially populations commonly under-represented in clinical trials.

## Conclusions

Our study shows that a complex multimodal lifestyle intervention with or without medical food is feasible in prodromal AD. Combining the lifestyle intervention with medical food may offer additional benefits on healthy dietary patterns, vascular risk burden, and cognitive-functional level. Larger, longer-term ongoing and planned multimodal intervention RCTs will provide more information on the feasibility, acceptability and effectiveness of personalized lifestyle intervention programs that target various factors related to brain health (e.g. the FINGER-NL trial testing a lifestyle + medical food combination, and other multimodal RTCs conducted within the World-Wide FINGERS network) [36]. Our findings also support future large-scale, multi-site RCTs investigating the effectiveness of multimodal lifestyle interventions, potentially combined with disease-modifying drugs in older adults with cognitive impairment.

### Supplementary Information

**Supplementary Material 1.**

## Data Availability

The MIND-AD consortium is open to requests from external researchers for data collected in the trial. Requesters will be asked to submit a study protocol, including the research question, planned analysis, and data required. The MIND-AD consortium will evaluate this plan (i.e., relevance of the research question, suitability of the data, quality of the proposed analysis, planned or ongoing MIND-ADmini analysis, and other matters) on a case-by-case basis and provide the data or reject the request. Shared data will encompass the data dictionary and de-identified participant data only. Any analysis will be conducted in collaboration with and on behalf of the MIND-AD consortium. Access is subject to the MIND-AD legal framework. An access agreement will be prepared and signed by both parties.
