# Supplementary material for: Integrating a multimodal lifestyle intervention with medical food in prodromal Alzheimer’s disease: the MIND-ADmini randomized controlled trial
Source: Alzheimers Res Ther. 2024 May 30;16:118. doi: 10.1186/s13195-024-01468-x (PMC11138035; doi:10.1186/s13195-024-01468-x)
Supplement: Supplementary file 1 — Supplementary Material 1. [file 13195_2024_1468_MOESM1_ESM.docx]

**SUPPLEMENTARY MATERIALS**

**S-Table 1. Baseline characteristics of specific factors included in FINRISK score**

|  | **Multimodal lifestyle intervention (n=32)** | **Multimodal lifestyle intervention+Medical food (n=31)** | **Control (regular care)**  **(n=30)** | **P-value** |
| --- | --- | --- | --- | --- |
| **Smoking status, n (%)** |  |  |  |  |
| No | 31 (96.88) | 30 (96.77) | 27 (90.00) |  |
| Currently | 1 (3.13) | 1 (3.23) | 3 (10.00) | 0.394 |
|  |  |  |  |  |
| **Systolic blood pressure*** | N=24 | N=25 | N=22 |  |
| Mean (SD) | 139.5 (15.42) | 145.4 (16.76) | 142.68 (19.99) | 0.461 |
|  |  |  |  |  |
| **Total Cholesterol** | N=32 | N=31 | N=29 |  |
| Median (min-max) | 4.9 (3.7-9.1) | 4.9 (2.8-9.3) | 4.6 (3-6.91) | 0.562 |
|  |  |  |  |  |
| **HDL** | N=32 | N=31 | N=29 |  |
| Median (min-max) | 1.61 (0.98-2.5) | 1.5 (0.77-2.8) | 1.6 (0.84-3.61) | 0.574 |
|  |  |  |  |  |
| **Diabetes, n(%)** |  |  |  |  |
| No |  |  |  |  |
| Yes | 1 (3.13) | 3 (9.68) | 4 (13.33) | 0.346 |

HDL=high-density lipoprotein

* Missing systolic blood pressure recordings at baseline were due to a technical issue at one of the sites in the beginning of the study.

**S-Table 2. Intervention adherence, and adherence to specific intervention domains**

|  | Multimodal lifestyle intervention  (n=32) | Multimodal lifestyle intervention+Medical food (n=31) | P-value |
| --- | --- | --- | --- |
| Overall intervention adherence, n (%) | 25 (78.1) | 27 (87.1) |  |
| Adherence to each intervention domain | |  |  |
| Nutrition |  |  |  |
| Group sessions, n (%) |  |  |  |
| 0/drop out | 7 (21.88) | 5 (16.13) |  |
| 1 | 3 (9.38) | 0 (0.00) |  |
| 2 | 2 (6.25) | 3 (9.68) |  |
| 3 | 20 (62.50) | 23 (74.19) | 0.292 |
| Individual sessions, n (%) |  |  |  |
| 0/drop out | 6 (18.75) | 6 (19.35) |  |
| 1 | 1 (3.13) | 0 (0.00) |  |
| 2 | 1 (3.13) | 0 (0.00) |  |
| 3 | 24 (75.00) | 25 (80.65) | 0.571 |
| Exercise |  |  |  |
| Attendance in the offered group-based gym sessions,^a^ median (IQR) | 0.79 (0.65—0.91) | 0.76 (0.68—0.87) | 0.984 |
| Cognitive training |  |  |  |
| Group sessions, n(%) |  |  |  |
| 0/drop out | 6 (18.75) | 3 (9.68) |  |
| 1 | 6 (18.75) | 1 (3.23) |  |
| 2 | 11 (34.38) | 14 (45.16) |  |
| 3 | 9 (28.13) | 13 (41.94) | 0.130 |
| In individual sessions, the completed proportion,^b^ median (IQR) | 0.56 (0.13—0.75) | 0.34 (0.17—0.73) | 0.897 |
| Vascular care |  |  |  |
| Attendance in the 3-month meetings with the study nurse | 26 (81.25) | 27 (87.10) | 0.525 |
| Attendance in the 6-month meetings with the study nurse | 30 (93.75) | 29 (93.55) | 0.974 |
| Medical food |  |  |  |
| Consuming bottles, median (IQR) | -- | 184 (180—185) | -- |

^a^ Calculated based on the number of sessions offered to each participant. During the 6-month trial, the amount of offered sessions could differ slightly due to public holidays and vacations.

^b^ In total 5 participants had missing data (automatic recordings).

IQR=Interquartile Range.

**S-Table 3. Detailed information for Figure 2**

|  | **Control group** | **Lifestyle group** | **Lifestyle + medical food group** |
| --- | --- | --- | --- |
| **Healthy dietary intake** |  |  |  |
| Marginal change (dy/dx) | -0.40 (-1.15, 0.35) | 0.71 (-0.02, 1.43) | 1.03 (0.30, 1.77) |
| Group×Time interaction | Reference | 1.11 (0.06, 2.15) | 1.43 (0.39, 2.48) |
| P-value for interaction | Reference | 0.038 | 0.007 |
| **Physical activity** |  |  |  |
| Marginal change (dy/dx) | -0.00 (-0.01, 0.00) | -0.00 (-0.01, 0.00) | 0.00 (-0.00, 0.01) |
| Group×Time interaction | Reference | -0.00 (-0.01, 0.01) | 0.00 (-0.00, 0.01) |
| P-value for interaction | Reference | 0.767 | 0.363 |
| **Cognitive and social activities** |  |  |  |
| Marginal change (dy/dx) | -1.63 (-3.86, 0.60) | -1.03 (-3.11, 1.05) | -0.50 (-2.62, 1.62) |
| Group×Time interaction | Reference | 0.60 (-2.45, 3.65) | 1.33 (-1.94, 4.21) |
| P-value for interaction | Reference | 0.698 | 0.470 |
| **Overall cardiovascular risk burden** |  |  |  |
| Marginal change (dy/dx) | -0.01 (-0.19, 0.16) | 0.02 (-0.14, 0.19) | -0.26 (-042, -0.09) |
| Group×Time interaction | Reference | 0.04 (-0.20, 0.27) | -0.24 (-0.48, -0.01) |
| P-value for interaction | Reference | 0.747 | 0.043 |
| **Overall healthy lifestyle** |  |  |  |
| Marginal change (dy/dx) | -0.13 (-0.71, 0.44) | 0.03 (-0.53, 0.59) | 0.42 (-0.15, 0.98) |
| Group×Time interaction | Reference | 0.16 (-0.64, 0.96) | 0.55 (-0.25, 1.36) |
| P-value for interaction | Reference | 0.687 | 0.179 |
